# Supplementary material for: Development of a complex multidisciplinary medication review and deprescribing intervention in primary care for older people living with frailty and polypharmacy
Source: PLoS One. 2025 Apr 22;20(4):e0319615. doi: 10.1371/journal.pone.0319615 (PMC12013936; doi:10.1371/journal.pone.0319615)
Supplement: S1 — (PDF) [file pone.0319615.s001.pdf]

Supporting file 1: MODIFY Qualitative study topic guides

Patient interview topic guide

**Interview questions**
We would like to understand people's experience in managing multiple medications and find ways to improve medication reviews offered by GP surgeries.<br><br>Can I start by asking you how do you manage your medications on a daily basis? What helps you to manage your medications? (alarms/reminders, carers, adherence aids)? What are the main challenges you experience in managing your medications?<br><br>Have you had your medications reviewed by a healthcare professional in your GP surgery?<br>If so, when was the last time you had your medications reviewed by a healthcare professional in your GP surgery? How did you feel about it? How often do you get your medications reviewed? What worked? What could have been done better?<br><br>What are your views about involving different healthcare professionals such as pharmacists, GPs and nurses in regularly reviewing patients' medications? Who do you think should be performing the review?<br><br>What would you like to happen in these reviews? And where do you think the medication review should take place?<br><br>What are your views about stopping medications if a healthcare professional decided that they are not needed anymore or could be harmful (e.g. causing side-effects)?
What sort of information do you think you would need to help you decide whether or not to stop a medication if it was found to not be needed anymore or harmful?<br><br>What would encourage you to stop a medication if it is not needed or harmful? (prompts might include relationship/trust with HCP, close follow-up, adequate information, enough support around patient…etc)<br><br>(If raised by the patient) What helps you to have trust in your health care professionals?<br><br>What helps you to have a good relationship with your health care professionals?<br><br>What would be the reasons for you to not reduce or stop a medication if it was found harmful or not needed? How do you think these worries could be addressed by your healthcare professionals?
What support would you like to have in place if you are asked to reduce or stop some of your medications if they are not needed or could be harmful?
Basic demographic questions:<br>Staff job role?<br>Years of experience?<br>Whether they are prescribers or not?<br>Gender?<br>Ethnicity?

Carer interview topic guide

Interview questions
We would like to understand patients and carers' experience in managing multiple medications and find ways to improve medication reviews offered by GP surgeries.<br><br>Can I start by asking you how do you manage your relatives' medications on a daily basis? What helps you to manage their medications? (alarms/reminders, carers, adherence aids)? What are the main challenges you experience in managing their medications?<br><br>Have they had their medications reviewed by a healthcare professional in your GP surgery?<br>If so, when was the last time they had their medications reviewed by a healthcare professional in your GP surgery? How did you/ they feel about it? How often do you get their medications reviewed? What worked? What could have been done better?<br><br>What are your views about involving different healthcare professionals such as pharmacists, GPs and nurses in regularly reviewing patients' medications? Who do you think should be performing the review?<br><br>What would you like to happen in these reviews? And where do you think the medication review should take place?<br><br>What are your views about your relative stopping medications if a healthcare professional decided that they are not needed anymore or could be harmful (e.g. causing side-effects)?
What sort of information do you think you and your relative would need to help you decide whether or not to stop a medication if it was found to not be needed anymore or harmful?<br>What would encourage you to stop one of your relatives' medication if it is not needed or harmful? (prompts might include relationship/trust with HCP, close follow-up, adequate information, enough support around patient…etc)<br><br>(If raised by the carer)<br>What helps you to have trust in their health care professionals?<br><br>What helps you to have a good relationship with their health care professionals?<br><br>What would be the reasons for you to not reduce or stop one of your relatives' medication if it was found harmful or not needed? How do you think these worries could be addressed by your healthcare professionals?
What support would you like to have in place if you are asked to reduce or stop some of their medications if they are not needed or could be harmful?
Basic demographic questions:<br>Age?<br>Gender?<br>Ethnicity?<br>Number of medications the patient is prescribed?<br>Their relationship with the patient?

Health care professional focus group/interview topic guide

Interview questions
The aim of the study is to design a multidisciplinary medication review in primary care focusing on identifying and deprescribing inappropriate medications among older people living with frailty.<br><br>Can I start by asking what are your views regarding carrying out medication reviews and deprescribing of inappropriate medications among older people living with frailty as a multidisciplinary team? Any advantages or disadvantages? Any challenges?<br><br>How would a multidisciplinary medication review, with the aim of deprescribing for older people living with frailty, best work within primary care? Who should be involved in the process? What are the main components of the process?<br>What are your views on carrying out a deprescribing medication review online vs in-person for older people living with frailty?<br><br>How is this approach to medication review different from what you already do in your practice?
What roles would different healthcare professionals have during a multidisciplinary deprescribing medication review process? Who do you think would be best placed to screen/identify patients for targeted medication review? Who should be performing the medication review?<br><br>What tools/resources are needed to facilitate reviewing medications and identifying inappropriate prescribing? Who should be following up patients? How often should patients medication be reviewed?<br><br>What type of medications would you feel most comfortable deprescribing for older people living with frailty? What medications you would be reluctant to deprescribe and why?<br><br>Can you identify any training or educational needs for you and your colleagues to enable you perform a deprescribing review? What are they? How could these needs best be met?
What would engage/ involve older patients living with frailty in the deprescribing medication review process?<br><br>What would facilitate good communication with older patients living with frailty and their carers/family members during a medication review process?<br><br>What would be the best way to communicate information and document decision between members of the multidisciplinary team? What should be in place to facilitate that? (Communication Performa/toolkit, weekly meetings to discuss cases, email management plan, and what should this include?)<br><br>What are the potential facilitators to integrating a multidisciplinary deprescribing medication review process for older people into your everyday practice?<br><br>What are the potential barriers to integrating a multidisciplinary deprescribing medication review process for older people into your everyday practice?
How best would older patients living with frailty be supported during the deprescribing medication review process? (information offered, shared-decision)

How best would older patients living with frailty be monitored/ followed-up in the deprescribing medication review process?

In your opinion, what are the indicators for the successful integration of the multidisciplinary medication review in your practice? how to measure that?

What would be the best way of evaluating/ measuring outcomes of a multidisciplinary deprescribing medication review process in primary care for older people living with frailty? (patient-outcomes, process-outcomes)

We are carrying out a review of studies on deprescribing interventions for older people with multidisciplinary team aspects and have developed some theories about how and why interventions may work or not work, and we'd like to put some of them to you for discussion please:

Role of the health care professional/ multidisciplinary team

a. If a health care professional (GPs, ANPs, Practice pharmacists) conducts a medication review in collaboration with a multidisciplinary team, and if there is good communication then they will feel more confident to take a decision to deprescribe because the responsibility will be shared as a team.

b. If practice pharmacists are involved in or lead medication reviews in primary care, then the intervention will be delivered to more older patients because this addresses the time constraints and workload challenges of GPs, and this has an effect on successful deprescribing interventions.

c. If GPs are involved in a medication review process in primary care, then the recommendations are more likely to be accepted and communicated to patients, as it is a multidisciplinary process. This has an effect on successful deprescribing interventions.

d. If primary care staff receive targeted training on deprescribing, then this will lead to higher rates of medication reviews and deprescribing because training could increase their skills, knowledge and confidence in managing and stopping inappropriate drugs.

e. If medication reviews focus on specific classes of drugs, then it would be more feasible for primary care teams to identify and stop inappropriate drugs and closely monitor the impact of stopping them.

f. If medication reviews focus on **targeting high-risk patients** (eg aged 65 years and over, taking 5 or more long term medications) then it would be more feasible for primary care teams to identify and stop inappropriate drugs and closely monitor the impact of stopping them.

Relationships and communication between health care professionals, patients, carers/ relatives

a. If health care professionals **involve and educate patients and carers/ relatives about the reasons for deprescribing/ make shared decisions with the patients and carers/ relatives about deprescribing** during a medication review, then patients will have a

better understanding of the reasons for deprescribing and feel more engaged in the deprescribing process. Therefore, they will be more likely to continue with any changes in medication leading to better self-efficacy/ better health outcomes.<br><br>b. If medication reviews focus on **patient preferences and priorities**, then patients would be engaged in in the process of a multi-disciplinary medication review and deprescribing. This will mean they are more likely to sustain any changes in their medication regime resulting in better self-efficacy/better health outcomes.<br><br>c. If there is **good communication and regular follow up** between health care professionals, patients and carers/ relatives, then patients will feel more engaged/ supported in the process of a medication review and deprescribing and be more likely to sustain any changes in their medication regime resulting in better self-efficacy/ better health outcomes.
Basic demographic questions:<br>Staff job role?<br>Years of experience?<br>Whether they are prescribers or not?<br>Gender? Ethnicity?
