# Supplementary material for: Development of a complex multidisciplinary medication review and deprescribing intervention in primary care for older people living with frailty and polypharmacy
Source: PLoS One. 2025 Apr 22;20(4):e0319615. doi: 10.1371/journal.pone.0319615 (PMC12013936; doi:10.1371/journal.pone.0319615)
Supplement: S2 — (PDF) [file pone.0319615.s002.pdf]

Evidence of deprescribing

You might ask yourself, what are the benefits of deprescribing and Are there any risks?

A lot of research has been done in the last 20 years to grow the evidence of deprescribing. Based on systematic reviews, deprescribing can potentially lead to:

- A reduction in the number of medications or inappropriate medications used by older people
- Resolution of adverse drug reactions and improved medication adherence
- No or small improvements in mortality or hospital admissions indicating safety of deprescribing
- Improvements in cognition and physical function (including frailty)
- Reduction in falls
- Reduction in drug costs
- Conflicting results in term of Quality of life

Stopping a medicines can obviously, potentially result in the return of the medical conditions or symptoms for which the medicine was initially prescribed. There can also be adverse drug withdrawal reactions with some medicines which will necessitate a gradual stopping and monitoring the process with a clear follow up plan.

Medication review and deprescribing as a shared process through a multidisciplinary team approach

Deprescribing of inappropriate medications is more effective it is done using a multidisciplinary team (MDT) approach. To facilitate this:

- Roles and responsibilities for each MDT member in relation to deprescribing need to be clearly defined.
- Foster mutual respect and trust between MDT members and opportunities for peer-learning and team training and development.
- Involve members of the team as appropriate based on their expertise, for example, involvement of social prescribers to offer non-pharmacological interventions.
- Have a clear and agreed process for communication between the team members in place. This can be done using the electronic tasks and messaging systems or face-to-face informal chat or team meetings.
- Document and share any medication changes and any follow up plans in the patient's record so all team members can access them and view agreed decisions.

MODIFY study

Engaging patients and carers in medication review and deprescribing decisions

To facilitate patient and carer engagement and trust:

- Prepare patients before the structured medication review (SMR) with written information on what to expect, the rationale for deprescribing and who may be carrying out their SMR, for example a practice pharmacist (see 'Reviewing your medicines' template letter).
- Involve family members or informal carers in the deprescribing process, as appropriate. For example, invite them to join the patient in the appointment or prompt patients to discuss medications with their informal carers before the appointment.
- During the SMR focus on patient's and carer's needs, preferences and goals (person-centred approach). *Ask* ***"what matters most to you?"***
- Clearly communicate to the patient the risks and benefits of continuing on a medicine. Resources such as GPEvidence.org and the PreQIPP Impact tool can help with this if prescribers are not sure of the risks and benefits.
- Involving patients and carers in deprescribing decisions has been shown to increase patient satisfaction, medication adherence, quality of life and well-being, and improve health outcomes

Identify deprescribing opportunities

Aim to educate and reach a shared understanding of the risks and benefits of medicines with tailored explanations. Ask:

Do you know why you are taking this medication?

Do you feel the medication is working?

Do you have any concerns about your medications?

Identify opportunities for deprescribing (stop, reduce dose, switch), this could include

high-risk medications (anticholinergics, opioids, fall-risk increasing drugs)

medications causing side effects (NSAIDs)

medications without clear indications or reported benefits to patients (statins, vitamins, PPI)

Recognise prescribing cascade

Consider using deprescribing tools such as the PresQIPP IMAPCT tool, medication appropriateness index

Talking about deprescribing

The majority of patients want to be involved with the decision-making process, to facilitate this:

- Start by agreeing to deprescribe one medicine at a time.
- Begin with ‘quick wins’, by starting with deprescribing changes tailored to the individual patient, taking their priorities into consideration, that could lead to noticeable improvements in symptoms, before progressing to deprescribing other medication.
- Offer deprescribing as a ‘drug holiday’, a temporary break from medication which can be monitored and restarted anytime if needed.
- Use the following questions to encourage engagement:

  What is the one medicine you feel you cannot live without?

  What is the one medicine you would rather not have to take?

  What are your main goals for this year? Lets adjust your medications to support these goals”

MODIFY study

Follow-up with patients and carers after a structured medication review

- Provide reassurance to the patient/ carer that they will be supported and monitored with any changes in their medications.
- Agree on a clear and robust plan for follow-up and monitoring of symptoms and provide the patient/ carer with a written copy (see NHS ‘safely stopping your medicine’ template provided).
- The follow-up plan should be tailored to the patient and may include a follow-up appointment or contact to review any changes, if necessary (for example a follow-up text, or phone appointment).
- Ensure the patient/ carer knows who to contact and how to contact them, should they have any concerns.
- Involve informal carers or family members in the follow-up process, as appropriate, for example by encouraging them to keep an eye on the patients' symptoms.
